# Supplementary material for: Chromothripsis during telomere crisis is independent of NHEJ, and consistent with a replicative origin
Source: Genome Res. 2019 May;29(5):737–49. doi: 10.1101/gr.240705.118 (PMC6499312; doi:10.1101/gr.240705.118)
Supplement: Supplemental Material [file supp_gr.240705.118_Supplemental_file_1.zip › contigs/annotated_contigs/DB81/contig.3.DB81_length_675_mean_cov_10.0.docx]

**DB81_length_675_mean_cov_10.0**

```
AGATATTATAACAGGTAGCAGGCTAGATTTGACCCATGGGGCATAGAGTTTGGTGACCCCAGCTATAAACTAGGAAGACAGCAGTAAAG
 >chr3:51692487-51692891 - E=2e-231
GGGGCTGGGTTCTAGACTAGGAAGAATCCAGTAAGGAGGCTGGGAATGGTGTTTTCTATGAGTCTGGGTATTTCATGGCACTTTTTCCC

TAACCTCAAAGGTACTCTAACCCAATAGAAGCTAGGGCAAAATCTCCTTACTGAACAAAAAGTCTCAAAGAGCCTCACACAATCATGGG

GCTTATTTAAAGGCTAATATGTAAAACAGGCTTGGATAGAAGACGTGGGTGCAGAAAAGAGGGGAAAAAAACAGGCTTAGATTGTGTCT

CAATACTGTTTAGCCCAGCTTTTTTATTCCACCAATCTCATATCCC|TC|CAGAATCTTTTTCAAAAGTGCCCTTCC|AGCTTCATTTG
                                               >chr3:51722532-51722561 + E=1e-05 p=2e-
GTGGCCAGG|TGGCCACTCCTGGCTTCTATCTGGCCACCCCTCTCCAATCCAGGCAGAGATGATCTGACAAGTCAGGAGCCCCTTGGCC
02        >chr3:51744373-51744597 - E=5e-123
GAGAGCCGATTTCACAGTTCCCCTCCACCACTGCCTTGAATGTGGCCCACCTCTCTCTGAGCCTTCGTTTCCTCATCTATAAAATGGGT

TAAAAATAATAGCAGCTAATATCAACGGTGCCCTTATGACTTGCCGGGCCCCTGAT
```
